# HS-GC–MS analysis of volatile organic compounds after hyperoxia-induced oxidative stress: a validation study

**DOI:** 10.1186/s40635-024-00600-3

**Published:** 2024-02-12

**Authors:** Thijs A. Lilien, Dominic W. Fenn, Paul Brinkman, Laura A. Hagens, Marry R. Smit, Nanon F. L. Heijnen, Job B. M. van Woensel, Lieuwe D. J. Bos, Reinout A. Bem, the DARTS study group, Alwin R. M. Verschueren, Tamara M. E. Nijsen, Inge Geven, Cristian N. Presură, Ronald Rietman, Marcus J. Schultz, Dennis C. J. J. Bergmans, Ronny M. Schnabel

**Affiliations:** 1 Department of Paediatric Intensive Care Medicine, Emma Children’s Hospital, Amsterdam UMC Location University of Amsterdam, Meibergdreef 9, Amsterdam, The Netherlands; 2 Laboratory of Experimental Intensive Care and Anaesthesiology, Amsterdam UMC Location University of Amsterdam, Meibergdreef 9, Amsterdam, The Netherlands; 3 Department of Pulmonary Medicine, Amsterdam UMC Location University of Amsterdam, Meibergdreef 9, Amsterdam, The Netherlands; 4 Department of Intensive Care Medicine, Amsterdam UMC Location University of Amsterdam, Meibergdreef 9, Amsterdam, The Netherlands; 5 Department of Intensive Care Medicine, Maastricht University Medical Centre+, Maastricht, The Netherlands; 6 Amsterdam Reproduction and Development Research Institute, Amsterdam UMC Location University of Amsterdam, Meibergdreef 9, Amsterdam, The Netherlands

**Keywords:** Hyperoxia, Oxidative stress, Volatile organic compounds, Mechanical ventilation, Intensive care unit, Headspace gas chromatography–mass spectrometry

## Abstract

**Background:**

Exhaled volatile organic compounds (VOCs), particularly hydrocarbons from oxidative stress-induced lipid peroxidation, are associated with hyperoxia exposure. However, important heterogeneity amongst identified VOCs and concerns about their precise pathophysiological origins warrant translational studies assessing their validity as a marker of hyperoxia-induced oxidative stress. Therefore, this study sought to examine changes in VOCs previously associated with the oxidative stress response in hyperoxia-exposed lung epithelial cells.

**Methods:**

A549 alveolar epithelial cells were exposed to hyperoxia for 24 h, or to room air as normoxia controls, or hydrogen peroxide as oxidative-stress positive controls. VOCs were sampled from the headspace, analysed by gas chromatography coupled with mass spectrometry and compared by targeted and untargeted analyses. A secondary analysis of breath samples from a large cohort of critically ill adult patients assessed the association of identified VOCs with clinical oxygen exposure.

**Results:**

Following cellular hyperoxia exposure, none of the targeted VOCs, previously proposed as breath markers of oxidative stress, were increased, and decane was significantly decreased. Untargeted analysis did not reveal novel identifiable hyperoxia-associated VOCs. Within the clinical cohort, three previously proposed breath markers of oxidative stress, hexane, octane, and decane had no real diagnostic value in discriminating patients exposed to hyperoxia.

**Conclusions:**

Hyperoxia exposure of alveolar epithelial cells did not result in an increase in identifiable VOCs, whilst VOCs previously linked to oxidative stress were not associated with oxygen exposure in a cohort of critically ill patients. These findings suggest that the pathophysiological origin of previously proposed breath markers of oxidative stress is more complex than just oxidative stress from hyperoxia at the lung epithelial cellular level.

**Supplementary Information:**

The online version contains supplementary material available at 10.1186/s40635-024-00600-3.

## Background

Oxygen therapy constitutes a cornerstone treatment in the intensive care unit, but may inadvertently cause hyperoxia-induced oxidative stress via increased formation of reactive oxygen species [1, 2]. The resulting enhanced oxidation of proteins, lipids and nucleotides leads to inflammation and ultimately cell death [1]. Overzealous use of oxygen has been linked to pulmonary injury and detrimental outcomes in preclinical and clinical studies [3–5]. Yet, it remains difficult to monitor hyperoxia-induced oxidative stress in clinical practice. Biomarkers of hyperoxia-induced oxidative stress could enable the early detection of pulmonary injury and tailor oxygen therapy.

Volatile organic compounds (VOCs) are abundant in exhaled breath and can be analysed noninvasively for diagnostic purposes [6]. This technique has garnered much interest over the past two decades and the process of biomarker identification in exhaled breath has been described in detail previously [7]. Various possible by-products of lipid peroxidation have been proposed as potential breath markers for oxidative stress, namely hydrocarbons, which have been reported to increase in healthy volunteers after hyperoxia exposure [8–13]. Whilst these VOCs are thought to arise from cellular lipids [9, 14], concerns persist about the heterogeneity of observed VOCs and their exact pathophysiological origin [15, 16]. It is unclear whether these VOCs originate from alveolar epithelial cells alone or from a more complex interaction mechanism with other cells present in the alveolar environment, i.e. immune cells, and bacteria. Preclinical studies that investigated the relationship between VOCs and oxidative stress so far used extracellular exposure to hydrogen peroxide (H_2_O_2_) and had conflicting results regarding observed VOCs [10, 17]. However, such a model does not necessarily reflect oxidative stress following hyperoxia exposure, as H_2_O_2_ concentrations used generally exceed physiologically representative levels and result in a very short exposure since H_2_O_2_ is decomposed within minutes [18, 19]. A more clinically representative model with hyperoxia-induced oxidative stress would thus be useful to improve breath marker validation within this specific context.

This study aimed to assess VOCs associated with the oxidative stress response in lung epithelial cells after exposure to hyperoxia by combining an in vitro model and headspace gas chromatography-mass spectrometry (HS-GC–MS). As a secondary aim, the diagnostic value of these VOCs was evaluated by examining their correlation with oxygen exposure in a cohort of critically ill patients.

## Materials and methods

### Cell culture

The current in vitro model was established as previously described in detail [17]. In brief, immortalised human alveolar basal epithelial (A549) cells (CCL-185) were cultured in Roswell Park Memorial Institute (RPMI) 1640 medium (Gibco, ThermoFisher Scientific, Waltham, MA, USA) supplemented with foetal bovine serum, penicillin–streptomycin, l-glutamine, gentamicin and amphotericin. Cells were incubated at 37 °C in 5% CO_2_ and passaged every 3–4 days until ~ 90% confluent. Before every experiment, cells from the culture flask were passaged by seeding ~ 1.5 × 10^5^ cells in 1 mL of supplemented RPMI-1640 in separate glass headspace vials (Markes International, Cincinnati, OH, USA) to eliminate plastic VOC contaminates [17, 20] and then incubated at 37 °C in 5% CO_2_ for 24 h until experimental exposure.

### Induction of oxidative stress

At the start of experimental exposure, the initially seeded medium was removed and replenished with 200 µL fresh supplemented RPMI-1640 medium. Three groups were created: air-exposed cells as normoxia controls, hyperoxia-exposed (~ 100% O_2_) cells as intervention, and 1 mM H_2_O_2_-exposed cells as oxidative stress-positive controls. Addition of H_2_O_2_ and airtight sealing of the vials was done as before [17]. The vials of hyperoxia-exposed cells were then purged with pure oxygen at a flow rate of 50 mL/min for 2 min using an air sampling pump (GSP-300FT-2; GASTEC) without disturbing the liquid interface, replacing the total vial volume (20 mL) five times. The vials remained airtight after purging and pilot experiments showed that the oxygen concentration was maintained after 24 h (~ 90% O_2_) by measuring the partial pressure of oxygen within the medium. The experimental setup to purge the vials is shown schematically in Additional file 1: Figure S1. All groups were incubated for 24 h in a purpose-built HiSorb™ agitator at 37 °C and 200 RPM (Markes International). The experiment was repeated on 4 different days.

### Cellular stress and cell death

Cytotoxicity was evaluated by markers of cellular inflammation (interleukin-8, IL8) and injury (lactate dehydrogenase, LDH), in the cell supernatant at the end of the 24-h exposure period. IL8 was measured using enzyme-linked immunosorbent assay per manufacturer’s instructions (R&D Systems Inc., Bio-Techne, Minneapolis, MN, USA) and LDH by a method developed by Zuurbier et al*.* [21]. Values of IL8 and LDH are expressed as a relative change from the mean levels in the removed supernatant before the start of the 24-h exposure period to account for differences between experimental days.

### Sampling of VOCs and sample processing

VOC sampling and processing was performed according to Fenn et al*.* [17]. After 22 h of exposure, high-capacity polydimethylsiloxane sorbent fibres (HiSorb™; Markes International, Cincinnati, OH, USA) were inserted through the vial caps and VOCs were captured from the headspace for 2 h. HiSorbs™ were removed after sampling and cleaned before transfer into empty desorption tubes (Markes International). HS-GC–MS analysis of HiSorb™ samples was performed within 5 days of collection. Further thermal desorption and HS-GC–MS processing methods are reported in Additional file 1: Methods S1.

### Clinical cohort

This was a secondary analysis of the DARTS (‘Diagnosis of Acute Respiratory disTress Syndrome’) study [22], a prospective, multicentre observational cohort study of critically ill patients with an expected duration of invasion ventilation > 24 h, in the Netherlands. Details on the clinical breath sampling, sample processing and patient characteristics of this cohort were reported before [22]. VOC data from breath samples collected on the first 2 days of invasive ventilation were included in the analysis. The current research expands on a prior study that examined oxygen exposure by the fraction of inspired oxygen (FiO_2_) [17]. However, in a clinical setting, hyperoxia-induced injury appears to be driven more by the partial arterial oxygen pressure (PaO_2_) rather than by FiO_2_ alone [23, 24]. Therefore, PaO_2_ was used to estimate oxygen exposure in patients in this repeat analysis and hyperoxia exposure was defined as a PaO_2_ > 16 kPa on the first measurement day [24].

### Statistical analysis

Before statistical analysis, noise removal of raw HS-GC–MS data, peak detection and peak alignment were performed using the ‘XCMS’ R-package [25]. VOC data were scaled by log10 transformation. Presence of a batch effect between experimental days was assessed by principal component analysis (PCA) and corrected using the ‘limma’ R-package [26]. Differences in IL8 and LDH between the three groups were evaluated by Dunn’s test for multiple comparisons with Holm’s adjustment.

For the primary analysis, both a targeted and non-targeted approach were used to identify oxidative stress-related VOCs. This approach was adapted from Fenn et al*.* [17]. Difference in VOC intensity between hyperoxia-exposed cells and controls was tested by Wilcoxon rank-sum test with adjustment for false discovery rate. A relevant change was defined as a twofold increase in the batch effect-corrected, unscaled median compared to the control group and an adjusted *P* value < 0.05. For the targeted approach, a gas standard (Massachusetts APH Mix, Supelco®; Supelco Inc., Bellefonte, PA, USA) was used to target specific VOCs that have previously been associated with hyperoxia exposure in healthy volunteers [8, 9, 12] amongst the sampled VOCs and evaluate their change (targets are reported in Additional file 1: Table S1). For the non-targeted approach, VOCs with a relevant change were selected for identification amongst the remaining VOCs. Subsequently, peaks on each retention time of interest were grouped in a retention time cluster and then identified using the National Institute of Standards and Technology library (NIST-library v.2.0a). Three criteria were used to define a valid identification to limit false discoveries: an NIST-match score ≥ 95, ≥ 3 peaks within a cluster [27], and a moderate correlation of the selected VOC with the rest of the cluster’s VOCs (Spearman’s *ρ* > 0.40). Following the in vitro model, all targeted VOCs and any identified non-targeted VOCs were searched within the VOC data of the clinical cohort and correlated with oxygen exposure. Quantile regression was used to visualise the median and quartile range of each VOC as a function of PaO_2_ on the first measurement day and correlation was tested by Spearman’s method. Intra-individual correlation of each VOC with PaO_2_ was assessed by repeated measures correlation [28]. In addition, the association between VOCs and hyperoxia exposure was tested by univariable logistic regression and the predictive value of each VOC to discriminate patients exposed to hyperoxia by area under the receiver operating characteristics curve (AUROCC) calculations. A sensitivity analysis defining hyperoxia by PaO_2_ > 20 kPa was included to evaluate the influence of the chosen threshold. All analyses were performed using R-4.3.2 (R Foundation for Statistical Computing, Vienna, Austria) with RStudio 2023.12.0 + 369 (RStudio, Boston, MA).

## Results

### Oxidative stress-induced cytotoxicity

**Fig. 1:**
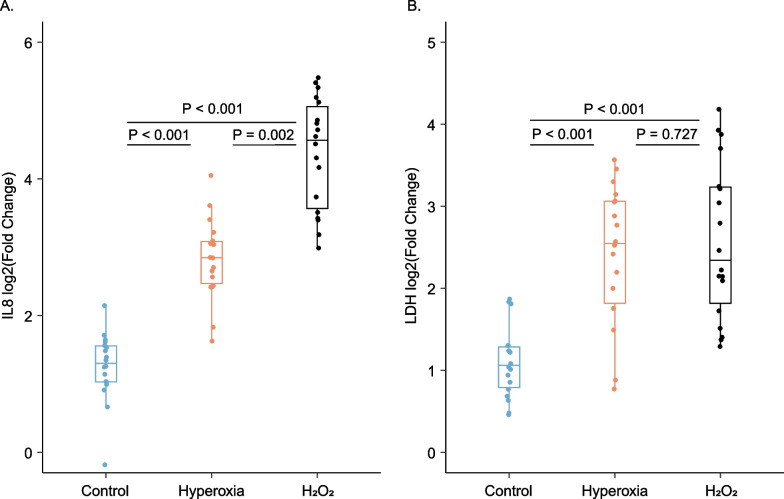
Hyperoxia-induced cytotoxicity of A549 cells. Relative fold change of interleukin-8 (**A**) and lactate dehydrogenase (**B**) supernatant concentrations of control, hyperoxia-exposed and H_2_O_2_-exposed cells in comparison to the levels before experimental exposure are shown. Differences were tested by Dunn’s test with Holm’s correction for multiple comparisons

Hyperoxia-exposed A549 cells (*N* = 18 vials) and H_2_O_2_-exposed cells (*N* = 18 vials) both revealed a significantly higher IL8 and LDH production after 24 h of exposure compared to controls (*N* = 18 vials), indicating a relevant degree of oxidative stress-induced cytotoxicity (Fig. 1). Hyperoxia-exposed cells showed a similar degree of cell injury as H_2_O_2_-exposed cells, but a significantly lesser degree of IL8 production.

### Targeted approach of VOCs linked to oxidative stress

After 24 h of exposure and data pre-processing, a total of 446 VOCs sampled in the headspace were included in the analysis. Visualisation by PCA plot showed heterogeneity between the 4 experimental days, which was sufficiently resolved after batch effect correction (Additional file 1: Figure S2).

**Fig. 2:**
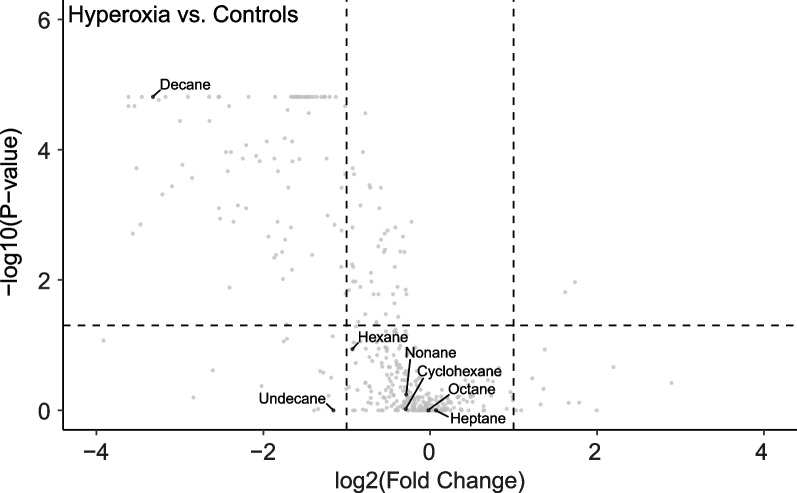
Targeted analysis of volatile organic compounds (VOCs) associated with oxidative stress. Volcano plot with the relative change of the median intensity of VOCs from the headspace of hyperoxia-exposed cells compared to controls is shown on the x-axis and the false discovery rate-adjusted P value on the y-axis. VOCs previously associated with oxidative stress that could be identified by gas standard are labelled

**Fig. 3:**
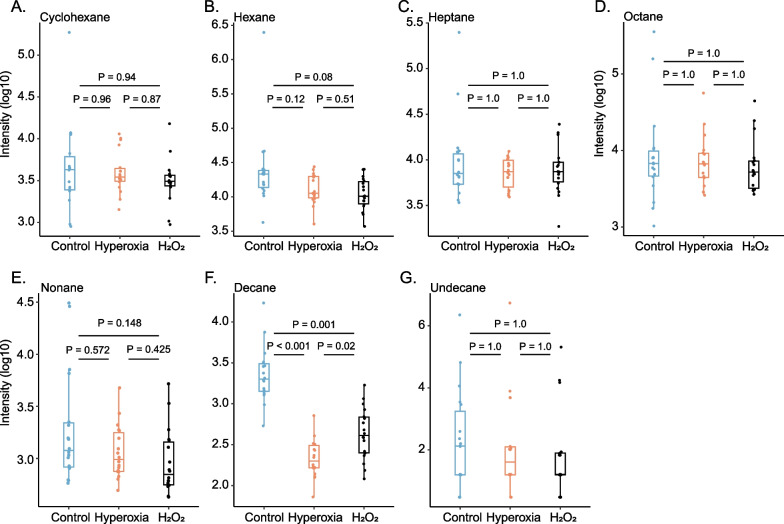
Target volatile organic compounds (VOCs) associated with oxidative stress. Intensities of identified target VOCs previously associated with oxidative stress per experimental condition, differences were tested by Wilcoxon rank-sum test with adjustment for false discovery rate

Seven target VOCs from the gas standard could be identified amongst the sampled VOCs and included: cyclohexane, hexane, heptane, octane, nonane, decane, and undecane. Despite signs of oxidative stress-induced cytotoxicity, none of these VOCs showed any significant increase after hyperoxia exposure compared with controls (Fig. 2). The same was true for H_2_O_2_-exposed cells (Fig. 3). In contrast, decane was significantly lower in hyperoxia-exposed cells and H_2_O_2_-exposed cells compared with controls (Fig. 3).

### Untargeted analysis of VOCs

**Fig. 4:**
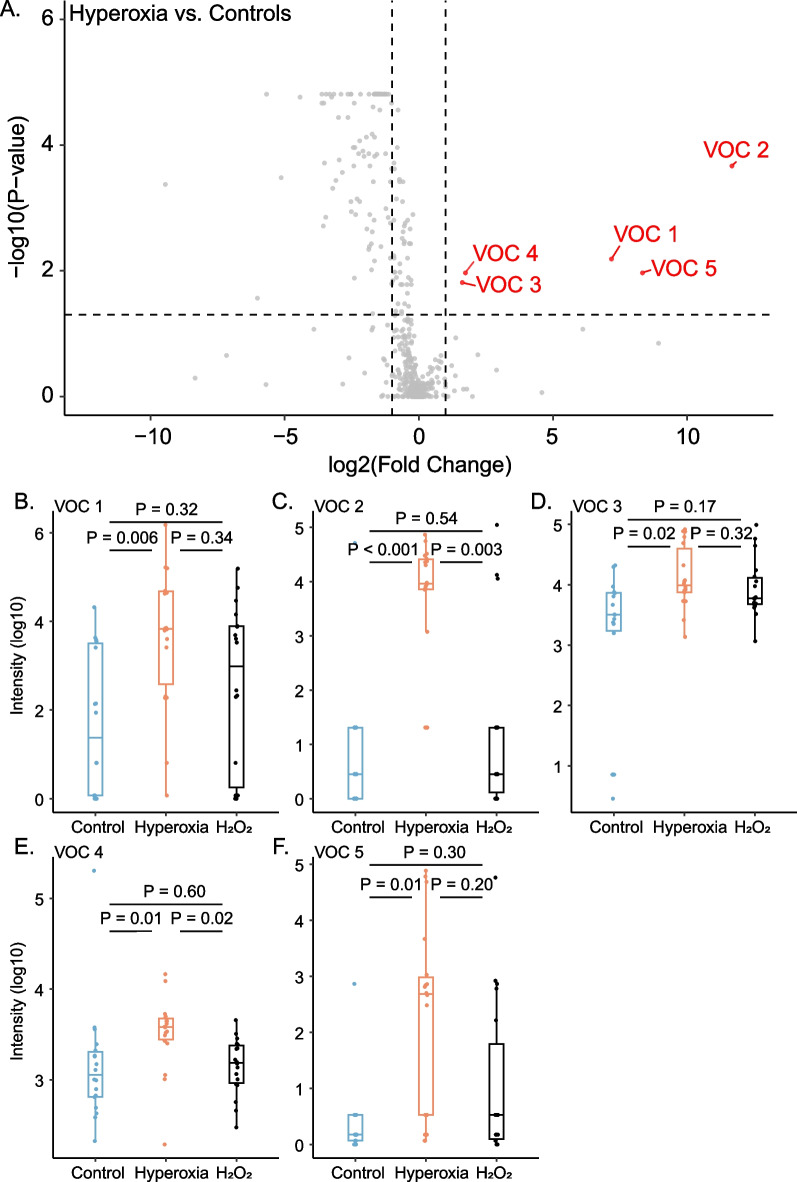
Untargeted analysis of volatile organic compounds (VOCs). Volcano plot (**A**) with the relative change of the median intensity of VOCs from the headspace of hyperoxia-exposed cells compared to controls is shown on the x-axis and the false discovery rate-adjusted P value on the y-axis. VOCs with at least a twofold increase of the median and an adjusted *P* value < 0.05 are labelled and their change per group is shown (**B**–**F**)

There were five VOCs which showed a relevant change after 24 h of exposure to hyperoxia (Fig. 4). Possible characteristics of these VOCs were alkenes, fatty alcohols, aldehydes, siloxanes, complex branched alkanes and straight-chain alkanes, but none of the VOCs met all criteria for valid identification (Additional file 1: Table S2). Post-hoc sensitivity omission of the twofold increase threshold criterion yielded no additional VOCs with a significant increase. Amongst the five unidentifiable VOCs, two were also significantly increased compared to H_2_O_2_-exposed cells. None of the selected VOCs showed any difference between H_2_O_2_-exposed cells and controls (Fig. 4).

### Correlation with oxygen exposure in patients

**Fig. 5:**
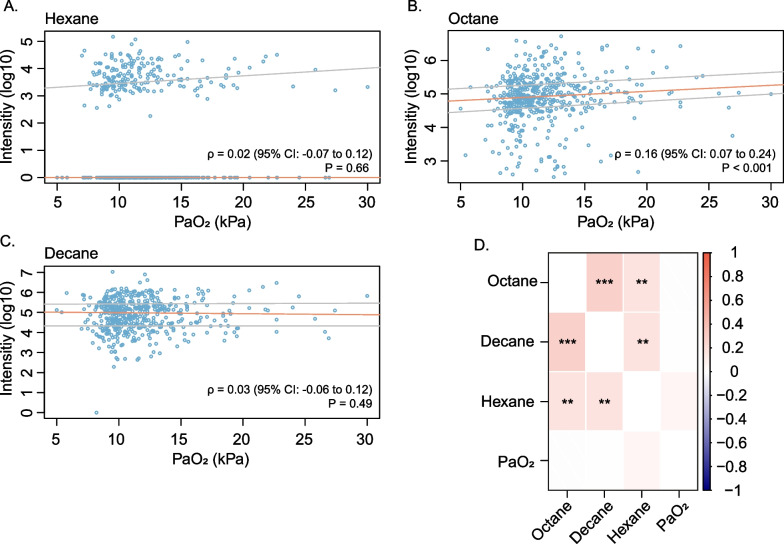
Correlation of volatile organic compounds (VOCs) and clinical oxygen exposure. Spearman’s correlation (*ρ*) of hexane (**A**), octane (**B**), and decane (**C**) with oxygen exposure in patients on the first measurement day is shown. Quantile regression was used to estimate the median (orange line) VOC intensity and interquartile range (grey lines) as a function of PaO_2_. Within subject correlation of VOCs and PaO_2_ over repeated measures is shown in D, 1 represents a strong positive correlation (red) and − 1 a strong negative correlation (blue). **, *P* < 0.01; ***, *P* < 0.001

Since the VOCs from the untargeted approach did not meet all identification criteria, they were not carried forward as additional targets to evaluate their association with oxygen exposure in a clinical setting. Three target VOCs could be identified within the cohort of critically ill patients, hexane, octane and decane. A total of 486 patients had available data on the target VOCs and oxygen exposure on measurement day 1. Octane correlated very weakly to weakly with PaO_2_ on the first measurement day, whereas hexane and decane showed no correlation (Fig. 5). Furthermore, none of the VOCs correlated with PaO_2_ within subjects (*N* = 289) over repeated measures (Fig. 5). There were 48 patients (9.9%) exposed to hyperoxia on the first measurement day. Octane was weakly associated with hyperoxia exposure and had a marginal discriminatory value with the AUROCC ranging from 0.51 to 0.68 (Table 1; Additional file 1: Figure S3). Hexane and decane were not associated with hyperoxia exposure and had no predictive value for discriminating patients exposed to hyperoxia (Table 1; Additional file 1: Figure S3). Last, the marginal discriminatory value of octane was lost when the threshold for hyperoxia exposure was further increased to > 20 kPa as a sensitivity analysis (Additional file 1: Figure S3).

**Table 1:** Association of VOCs linked to oxidative stress with hyperoxia exposure in patients

VOC log10 (intensity)	OR (95% CI)^a^	*P* value	AUROCC (95% CI)^b^
Hexane	1.15 (0.99 to 1.35)	0.07	0.56 (0.49 to 0.64)
Octane	1.57 (1.00 to 2.45)	0.05^c^	0.60 (0.51 to 0.68)
Decane	1.20 (0.82 to 1.76)	0.35	0.48 (0.39 to 0.57)

Hyperoxia exposure was defined as a partial arterial oxygen pressure > 16.0 kPa

*VOC* volatile organic compound, *OR* odds ratio, *CI* confidence interval, *AUROCC* area under the receiver operating characteristics curve

^a^Per log10 increment

^b^Predictive value with hyperoxia exposure as outcome

^c^Rounded up

## Discussion

This study failed to find evidence that VOCs previously linked to oxidative stress and hyperoxia exposure increase as a result of hyperoxia-induced oxidative stress in lung epithelial cells. Furthermore, VOCs potentially indicative of hyperoxia exposure could not be identified with sufficient certainty to rule out false discoveries. VOCs previously associated with oxidative stress also showed no clinically relevant association with oxygen exposure or the occurrence of hyperoxia within a cohort of critically ill patients.

The observed oxidative stress-induced cytotoxicity and dynamics of targeted VOCs were consistent between hyperoxia and H_2_O_2_-exposed cells, including the decrease in decane compared to controls. It is unclear why decane was lower after oxidative stress exposure, but similar trends with oxidative stress have been observed for octane in two previous in vitro studies [17, 20]. There is a possibility that, instead of increased production from lipid peroxidation, oxidative stress resulted in autoxidation of these alkanes. This remains a relatively improbable reaction at body temperature, however, and would require free radical chain initiators or other catalysts [29, 30]. Interestingly, studies with healthy volunteers have consistently found an association of oxygen exposure with an increase in various alkanes, methyl alkanes and aldehydes, although they remain heterogeneous in terms of specific VOCs found [8, 11, 12]. These studies vary largely in exposure dose, ranging from hyperbaric hyperoxia, where the partial pressure of oxygen exceeds atmospheric pressure, to a study exposing subjects for merely 30 min to 28% oxygen via nasal prongs [8, 12]. It is highly unlikely that the latter exposure would have resulted in hyperoxia-induced injury [3, 4], but evidence from a swine model suggests that increases in breath markers of oxidative stress may precede relevant injury [31]. Therefore, it is particularly intriguing that in the current in vitro model, prolonged exposure to a very high dose of oxygen did not result in an increase of alkanes, whilst cellular inflammation and injury were evident. The current findings indicate that the biosynthetic origin of targeted VOCs, which were previously proposed as breath markers of hyperoxia-induced oxidative stress in healthy volunteers [8, 11, 12], is not simply the occurrence of oxidative stress at the local lung epithelial cellular level. Hypothetically, a more complex mechanism, such as interaction between immune cells and the alveolar cells or altered bacterial metabolism, may be involved in the production of these VOCs [32].

The targeted analysis included only alkanes, but hyperoxia exposure of lung epithelial cells did not yield any other distinct breath markers that could be identified with sufficient certainty either. The fact that most untargeted VOCs were detected at retention times of more than 15 min made identification difficult. Co-elution becomes increasingly problematic at higher retention times and is difficult to distinguish with one-dimensional GC–MS [33]. Nonetheless, the absence of other VOCs after hyperoxia exposure suggests that, in addition to more complex mechanisms which may underlie VOC production, the possibility of false discoveries by chance or spurious correlations should not be overlooked [16, 20]. The large number of VOCs present in headspace and breath samples usually far outnumber the study cases, which increases the risk of false positives from untargeted multiple testing. This was further highlighted by the recent DARTS-study, which found no benefit of octane as a predictor for the diagnosis of acute respiratory distress syndrome in a large-scale validation study, despite the promising results of the earlier discovery study [22, 34].

Whilst the relative simplicity of the in vitro model might partly explain the observed negative findings, this is different for the complex in vivo setting of the analysed clinical cohort. A previous study that analysed the same cohort did not find an association of the identified alkanes with oxygen exposure when defined by FiO_2_ [17]. In line with these findings, none of the alkanes that were previously associated with hyperoxia exposure or oxidative stress correlated clearly with oxygen exposure or had relevant diagnostic value in distinguishing patients with or without hyperoxia exposure when defined by PaO_2_. This finding contrasts with the studies that used healthy volunteers and challenges the validity of the proposed breath markers [8, 11, 12]. However, critically ill patients differ greatly from healthy volunteers and, besides hyperoxia, there are a number of sources that may amplify oxidative stress, for example immune cell activity, concurrent pulmonary infection, and lung stretch from invasive ventilation [35–37]. The association of VOCs with hyperoxia exposure could potentially be obscured by the contribution of other oxidative stress-inducing factors in such patients, which, if true, would have serious implications for the clinical application of these VOCs as biomarkers. If these VOCs could merely provide a general estimate of the oxidative status, using them to tailor oxygen therapy becomes complicated. It then remains unclear whether any shifts in VOCs reflect changes in supportive care or that of other underlying factors.

This study was strengthened by its translational approach using one of the largest multicentre cohorts with VOC data currently available for the validation of volatile metabolites both in vitro and in vivo [22]. The use of glass airtight culture vials minimised the potential effect of contaminates on compound discovery. In addition, the implemented strict identification criteria and the use of an external gas standard limited the risk of false discoveries. The study also has limitations. The in vitro model with a monolayer of A549 cells is not the best representation of the alveolar epithelial environment. It could be worthwhile repeating this model with other cell types, such as endothelial cells or differentiated alveolar cells, and to include immune cells or bacteria to strengthen the robustness of the current findings. Air–liquid interface culture models have previously been used to better mimic the alveolar compartment [38]. Unfortunately, these models often still involve the use of plastics, increasing the risk of erroneous results through unwanted contaminates [16, 20]. Organoids may also be an interesting alternative to better mimic the in vivo setting [39]. The use of one-dimensional GC–MS analysis, despite being similar to previous studies with healthy volunteers [8], may have led to missed observations of potential VOCs. In addition, ethane and pentane, known by-products of lipid peroxidation [9, 11, 13], could not be captured due to the chromatographic method employed. Conclusions on the association of these specific compounds with oxygen exposure should therefore not be inferred from this study.

## Conclusions

This translational study failed to find evidence that hyperoxia-induced oxidative stress in alveolar epithelial cells results in the increase of alkanes previously associated with hyperoxia exposure or any other identifiable volatile metabolite. Moreover, hyperoxia exposure in a large clinical critically ill patient cohort showed no relevant association with volatile metabolites previously linked to hyperoxia exposure. These findings highlight the need for validation of the pathophysiological origin of VOCs previously associated with hyperoxia or oxidative stress before we interpret them as possible biomarkers.

### Supplementary Information

**Additional file 1: Methods S1. Table S1.** Targeted Volatile Organic Compounds from gas standard. **Table S2.** Identification criteria in untargeted analysis. **Figure S1.** Schematic graphic of hyperoxia exposure. **Figure S2.** Batch-effect correction of experimental days. **Figure S3.** Discriminatory value of Volatile Organic Compounds for hyperoxia exposure. **Figure S4.** Distribution of VOCs and PaO_2_ within the clinical cohort.

## Data Availability

The datasets used and/or analysed during the current study are available from the corresponding author on reasonable request.

## Abbreviations

VOCs: Volatile organic compounds
H_2_O_2_: Hydrogen peroxide
HS-GC–MS: Headspace gas chromatography and mass spectrometry
A549: Immortalised human alveolar basal epithelial cells
RPMI: Roswell Park Memorial Institute
IL8: Interleukin-8
LDH: Lactate dehydrogenase
FiO_2_: Fraction of inspired oxygen
PaO_2_: Partial arterial oxygen pressure
PCA: Principle component analysis
AUROCC: Area under the receiver operating characteristics curve
