# Supplementary material for: HS-GC–MS analysis of volatile organic compounds after hyperoxia-induced oxidative stress: a validation study
Source: Intensive Care Med Exp. 2024 Feb 12;12:14. doi: 10.1186/s40635-024-00600-3 (PMC10861410; doi:10.1186/s40635-024-00600-3)
Supplement: Supplementary file 1 — Additional file 1: Methods S1. Table S1. Targeted Volatile Organic Compounds from gas standard. Table S2. Identification criteria in untargeted analysis. Figure S1. Schematic graphic of hyperoxia exposure. Figure S2. Batch-effect correction of experimental days. Figure S3. Discriminatory value of Volatile Organic Compounds for hyperoxia exposure. Figure S4. Distribution of VOCs and PaO2 within the clinical cohort. [file 40635_2024_600_MOESM1_ESM.docx]

**Additional Content to:**

Volatile Organic Compounds Identified After Hyperoxia-Induced Oxidative Stress: a Validation Study by *Lilien et al.*

**INDEX**

| **Content** | **Pg.** |
| --- | --- |
| **Methods S1** | 2 |
| **Table S1.** Targeted Volatile Organic Compounds from gas standard | 3 |
| **Table S2.** Identification criteria in untargeted analysis | 4 |
| **Figure S1.** Schematic graphic of hyperoxia exposure | 5 |
| **Figure S2.** Batch-effect correction of experimental days | 6 |
| **Figure S3.** Discriminatory value of Volatile Organic Compounds for hyperoxia exposure | 7 |
| **Figure S4.** Distribution of VOCs and PaO_2_ within the clinical cohort | 8 |
| **References** | 9 |

**Methods S1**

Thermal desorption – Gas chromatography – Mass Spectrometry (TD GC-MS)

# As described by Fenn et al. [1], sorbent tubes were heated to 250 ℃ for 5 min with a flow of 30 mL/min (Markes TD100 autosampler and desorber [Cincinnati, Ohio, USA]). VOCs were captured on a cold trap at 25 ℃ and re-injected by rapidly heating the trap to 280 ℃ for one minute. VOCs were injected splitless through a transfer line at 180 ℃ onto an Inertcap 5MS/Sil GC column [30 m, ID 0.25 mm, film thickness 1 μm, 1,4-bis(dimethylsiloxy)phenylene dimethyl polysiloxane (Restek, Breda, The Netherlands)] with a flow of 1.2 mL/min. Oven temperature was kept isothermal at 40 ℃ for 5 minutes, then increased to 280 ℃ at 10 ℃/min and kept isothermal at 280 ℃ for 5 minutes. Molecules were ionized using electron ionization (70 eV), and the fragment ions were detected using a quadrupole mass–spectrometer (GCMS–GP2010, Shimadzu, Den Bosch, the Netherlands) with a scan range of 37–300 Da.

**Table S1. Volatile Organic Compounds Associated with Hyperoxia-Induced Oxidative Stress Targets in Gas Standard**

| **Compound** | **CAS No.** | **Mol. Formula** | **Ret. time range (sec)** | **Reference** |
| --- | --- | --- | --- | --- |
| Cyclohexane | 110-82-7 | C6H12 | 358-375 | [2] |
| Hexane | 110-54-3 | C6H14 | 234-250 | [2-4] |
| Heptane | 142-82-5 | C7H16 | 430-436 | [3, 4] |
| Octane | 111-65-9 | C8H18 | 600-618 | [2-4] |
| Nonane | 111-84-2 | C9H20 | 760-770 | [2, 4] |
| Decane | 124-18-5 | C10H22 | 902-907 | [2, 4] |
| Undecane | 1120-21-4 | C11H24 | 1023-1029 | [2, 4] |
| 2,3-dimethylpentane | 565-59-3 | C7H16 | 352-360 | [2]* |
| 2,3-dimethylheptane | 3074-71-3 | C9H20 | 697-707 | [2]* |

*Exact compound has not been reported, but a similar dimethyl alkane

**Table S2. Identification of Volatile Organic Compounds from Untargeted Analysis**

| **VOC ID** | Characteristics of compound | **Ret. time (sec)** | **No. VOCs on ret. time** | **Correlation ID^a^** | **NIST match** | **No. criteria met** |
| --- | --- | --- | --- | --- | --- | --- |
| VOC1 | Straight-chain alkene; 10-14 carbon molecules (C)  Fatty alcohol; C10-C12 | 1122 | 15 | 0.10 | Yes | 2/3 |
| VOC2 | Aldehyde; C8-C12 | 907 | 11 | 0.15 | Yes | 2/3 |
| VOC3 | Siloxane | 645 | 2 | 0.61 | Yes | 2/3 |
| VOC4 | Branched alkanes (methyl/propyl/butyl groups); C13-C14  Straight-chain alkane; C14-C21 | 1245 | 1 | NA | No | 0/3 |
| VOC5 | Siloxane | 886 | 2 | 0.69 | No | 1/3 |

Criteria for certain identification were: cluster of ≥3 VOCs on ret. time, ≥0.40 correlation of ID VOC with other VOCs on cluster, and a match score ≥95 in the NIST library. a, Spearman correlation coefficient. VOC, volatile organic compound

**Figure S1.** Schematic graphic of hyperoxia exposure

A. Air sampling bag with pure oxygen (Tedlar bag); B. Sealed headspace vial with A549 cells in RPMI-1640 medium; C. Air sampling pump (GSP-300FT-2; GASTEC). A constant flow of 50 mL/min is maintained by the pump and the headspace vial is flushed with pure oxygen from the bag for 2 minutes, replacing the total volume of the vial (20mL) five times. After 24 hours of incubation the oxygen concentration within the vial was still approximately 90%.


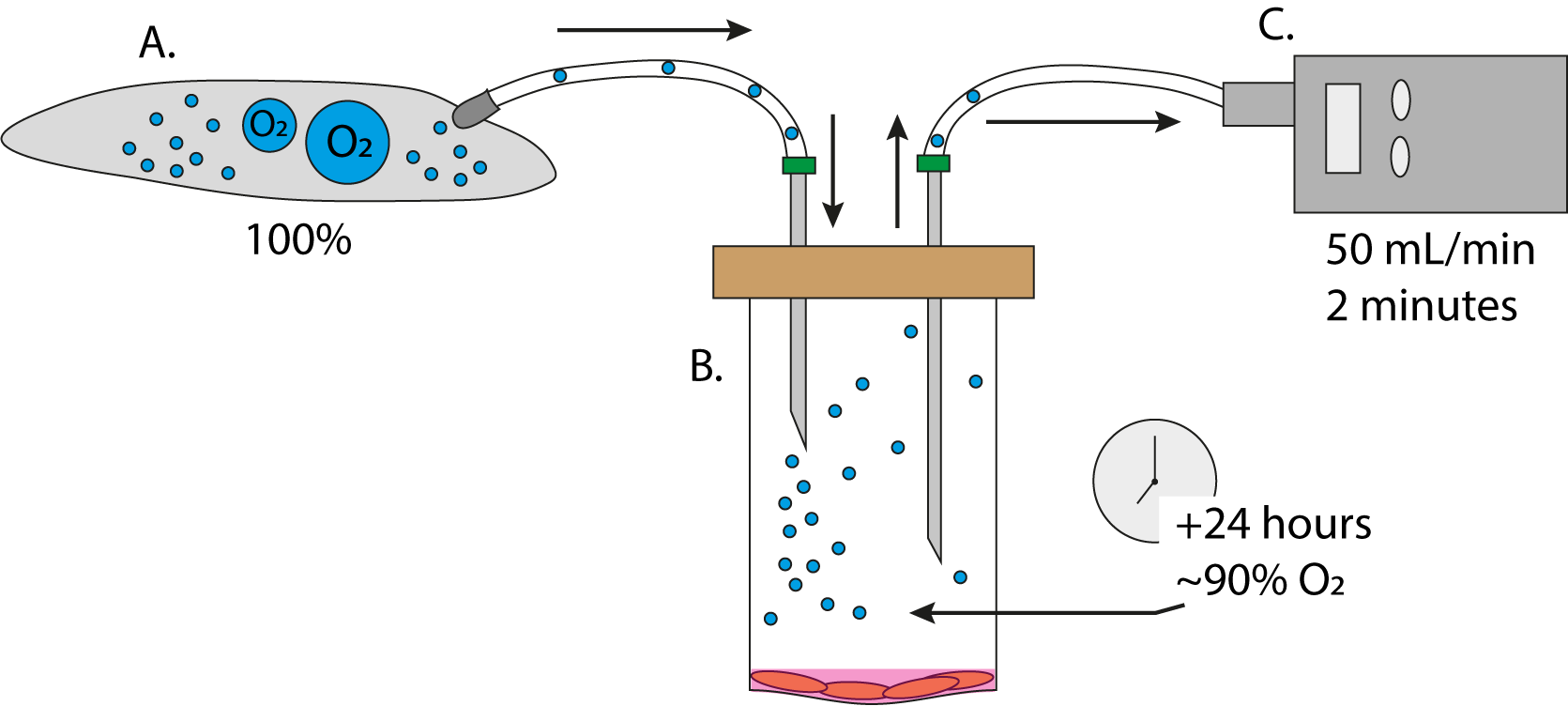


**Figure S2.** Batch-effect correction of experimental days


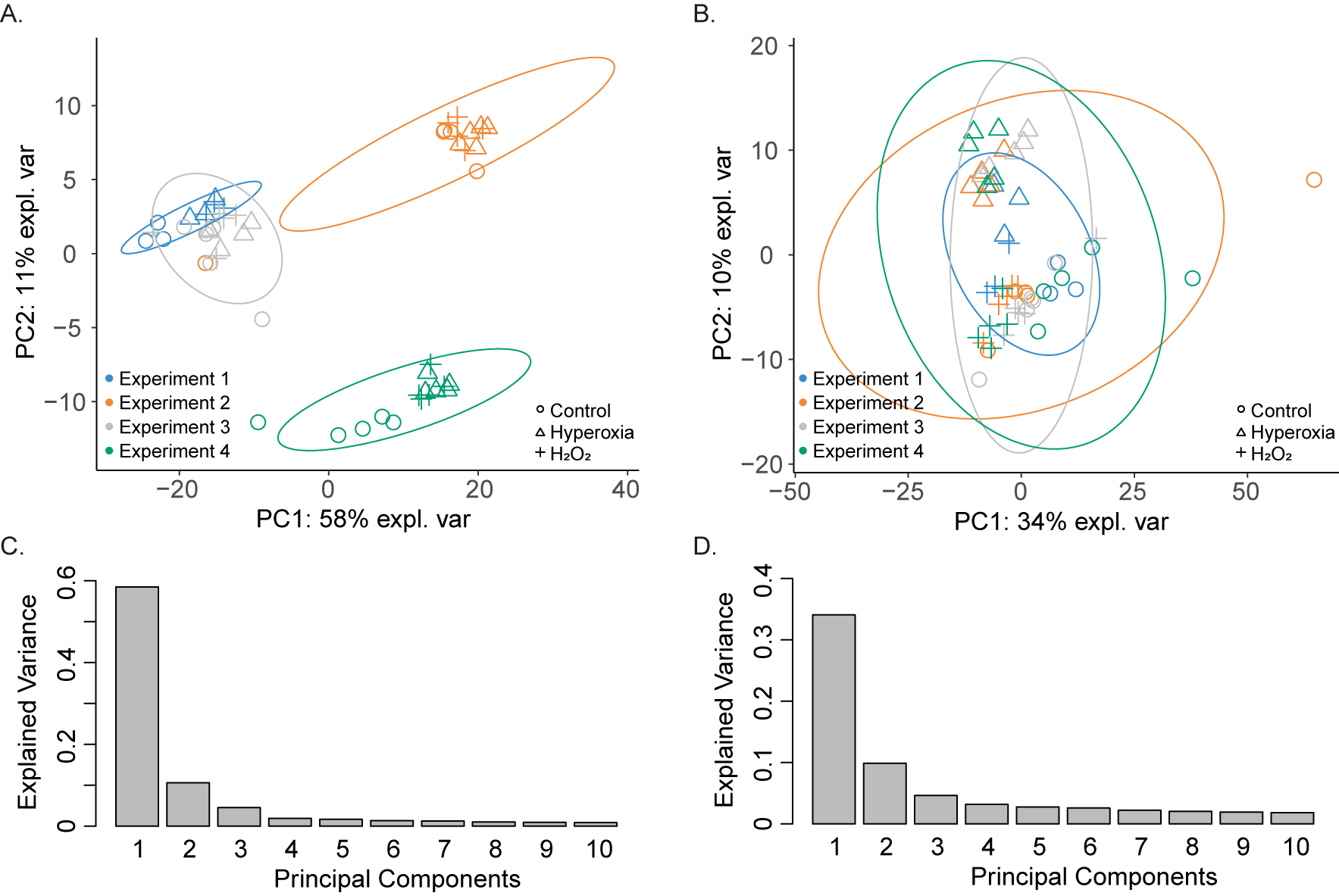


**Figure S3.** Receiver Operating Characteristics of Volatile Organic Compounds and Hyperoxia Exposure


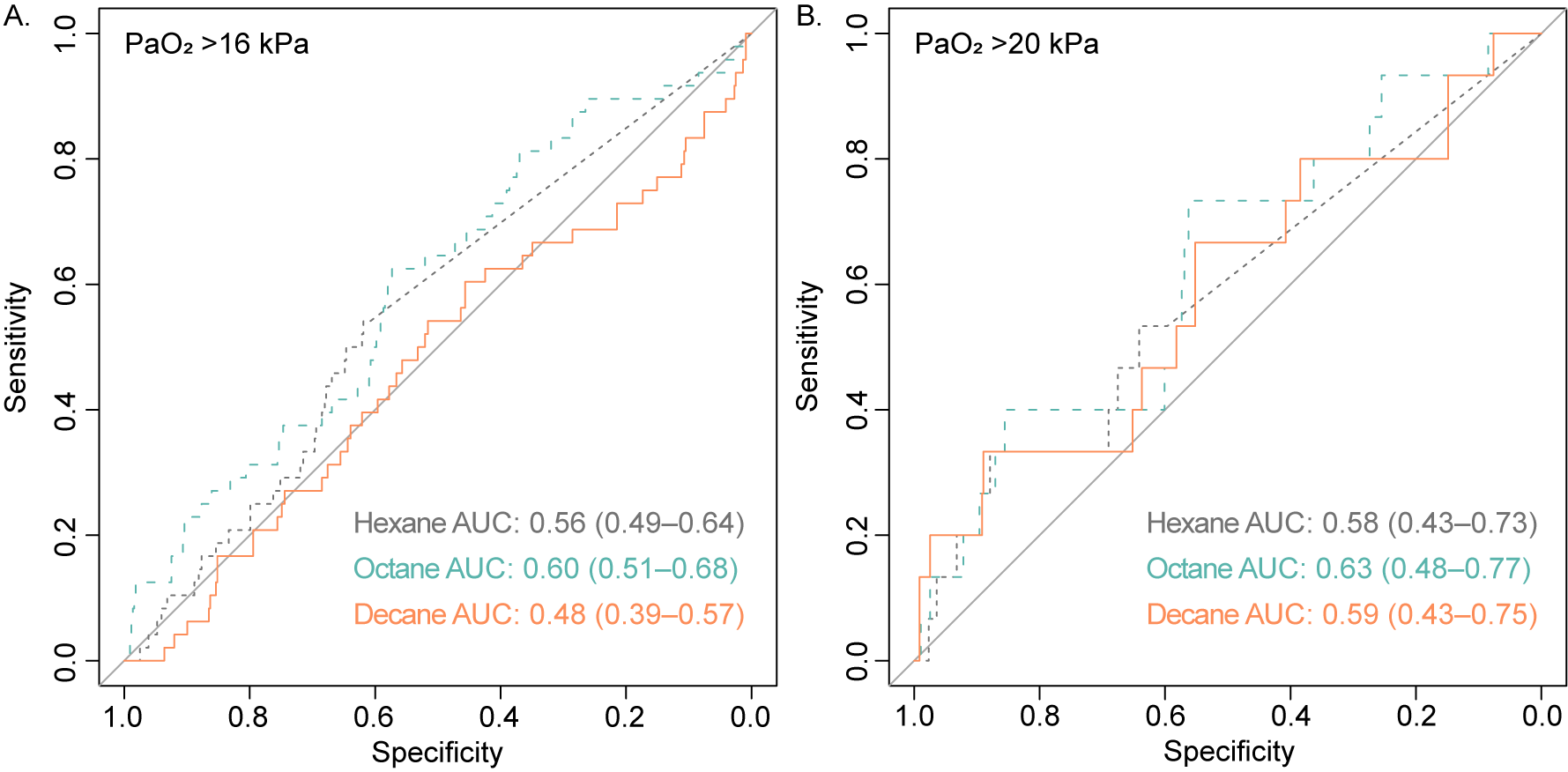


Area under the receiver operating characteristics curves are calculated with its 95% confidence interval to estimate the discriminatory value of each VOC. Hyperoxia was defined by a partial arterial oxygen pressure (PaO_2_) > 16 kPa (A) or > 20 kPa as alternative threshold (B) at the first measurement day in the DARTS-cohort.

**Figure S4.** Distribution of VOCs and PaO_2_ within the clinical cohort

Individual data points are shown by the dots with each box representing the median and interquartile range.


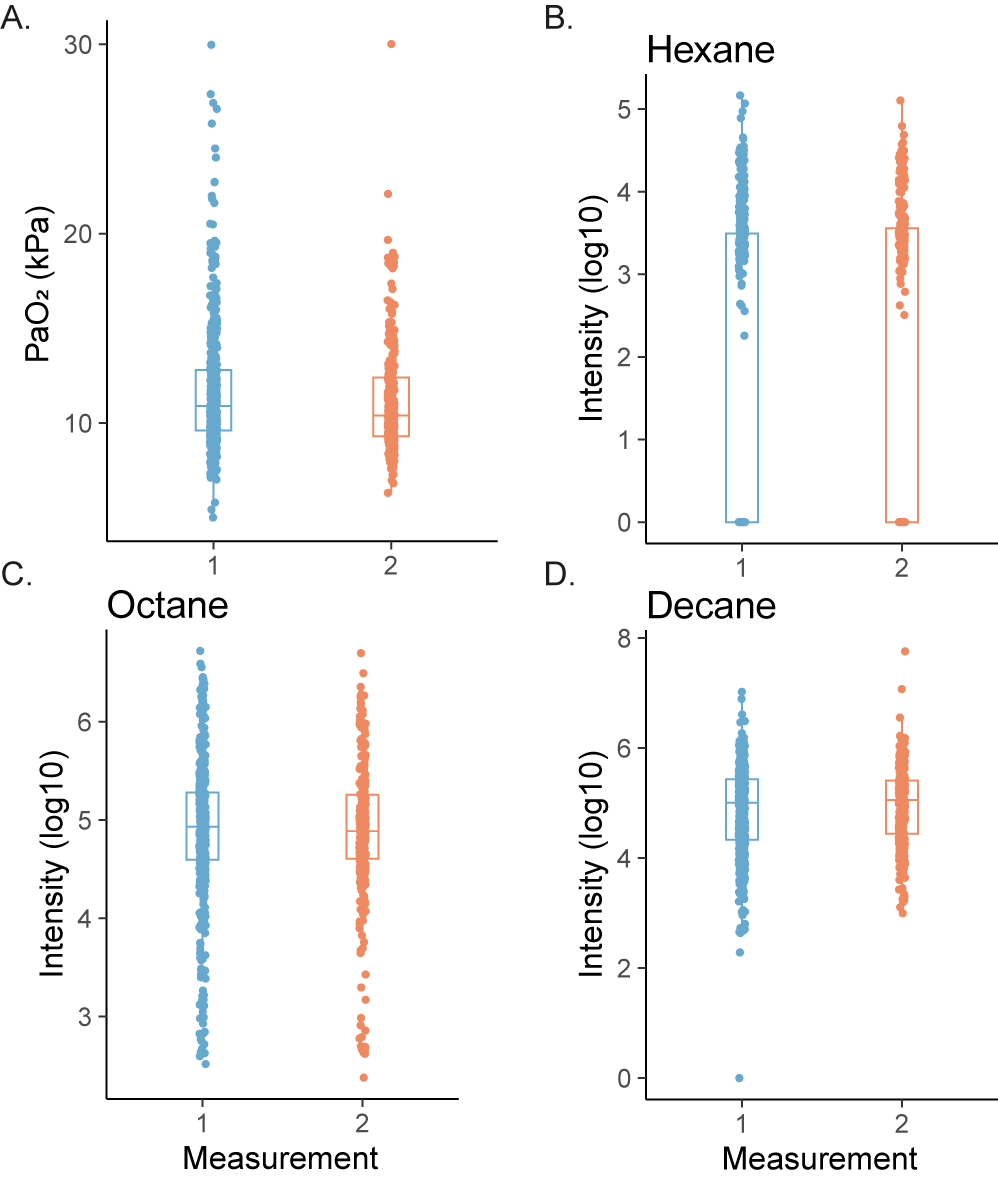


**References**

1. Fenn D, Lilien TA, Hagens LA, Smit MR, Heijnen NFL, Tuip-de Boer AM, et al. (2023) Validation of volatile metabolites of pulmonary oxidative injury: a bench to bedside study. ERJ Open Res 9(2). <https://doi.org/10.1183/23120541.00427-2022>

2. de Jong FJM, Brinkman P, Wingelaar TT, van Ooij PAM, van Hulst RA (2022) Volatile Organic Compounds Frequently Identified after Hyperbaric Hyperoxic Exposure: The VAPOR Library. Metabolites 12(5). <https://doi.org/10.3390/metabo12050470>

3. Kneepkens CM, Lepage G, Roy CC (1994) The potential of the hydrocarbon breath test as a measure of lipid peroxidation. Free Radic Biol Med 17(2):127-60. <https://doi.org/10.1016/0891-5849(94)90110-4>

4. Phillips M, Cataneo RN, Greenberg J, Grodman R, Gunawardena R, Naidu A (2003) Effect of oxygen on breath markers of oxidative stress. Eur Respir J 21(1):48-51. <https://doi.org/10.1183/09031936.02.00053402>
